# Copper-cysteamine nanoparticles in cancer treatment: a systematic review

**DOI:** 10.1007/s10565-025-10103-w

**Published:** 2025-11-27

**Authors:** Mahsa Ejtema, Nahid Chegeni, Britta Langen, Mousa Ahmadi Marallu, Zeinab Shafiei Seifabadi, Omid Azadbakht, Mohammadreza Nazarian, Diana Spiegelberg, Marcin Kruszewski

**Affiliations:** 1 Department of Surgical Sciences, Uppsala University, Uppsala, Sweden; 2 Department of Medical Physics, School of Medicine, Ahvaz Jundishapur University of Medical Sciences, Ahvaz, Iran; 3 Advanced Imaging Research Center, University of Texas Southwestern Medical Center, Dallas, TX USA; 4 Department of Radiation Oncology, University of Texas Southwestern Medical Center, Dallas, TX USA; 5 Department of Cell and Molecular Biology, Faculty of Life Sciences and Biotechnology, Shahid Beheshti University, Tehran, Iran; 6 Department of Anatomical Sciences, School of Medicine, Yasuj University of Medical Sciences, Yasuj, Iran; 7 Student Research Committee, Ahvaz Jundishapur University of Medical Sciences, Ahvaz, Iran; 8 Centre for Radiobiology and Biological Dosimetry, Institute of Nuclear Chemistry and Technology, Warsaw, Poland; 9 Department of Molecular Biology and Translational Research, Institute of Rural Health, Lublin, Poland

**Keywords:** Copper-cysteamine nanoparticles, Photodynamic therapy, X-ray induced photodynamic therapy, Cancer, Reactive oxygen species, Nanomedicine

## Abstract

Copper-cysteamine nanoparticles (Cu-Cy NPs) represent an innovative approach for cancer therapy due to their unique ability to be activated by multiple physical and chemical stimuli. This review systematically evaluates studies investigating Cu-Cy NPs in combination with chemical agents and diverse energy sources, including X-rays, UV light, microwaves, and ultrasound. A comprehensive literature search in PubMed, Scopus, and Web of Science up to August 2025 identified 18 relevant studies encompassing both in vitro and in vivo experiments. Across these studies, Cu-Cy NPs consistently suppressed tumor growth and triggered cancer cell death by generating reactive oxygen species (ROS) and enhanced therapeutic effects when combined with co-treatments such as disulfiram, potassium iodide, and other adjunct therapies. The multi-modal activation of Cu-Cy NPs, along with their ability to enhance existing therapeutic approaches, demonstrates a novel strategy in cancer treatment that integrates chemical and physical mechanisms for maximal efficacy. These findings underscore the nanoparticles’ potential to transform current oncology strategies, offering targeted, versatile, and personalized therapeutic options. Continued investigation is essential to fully elucidate their mechanisms, optimize treatment protocols, and translate these promising preclinical results into safe and effective clinical applications.

**Graphical Abstract:**

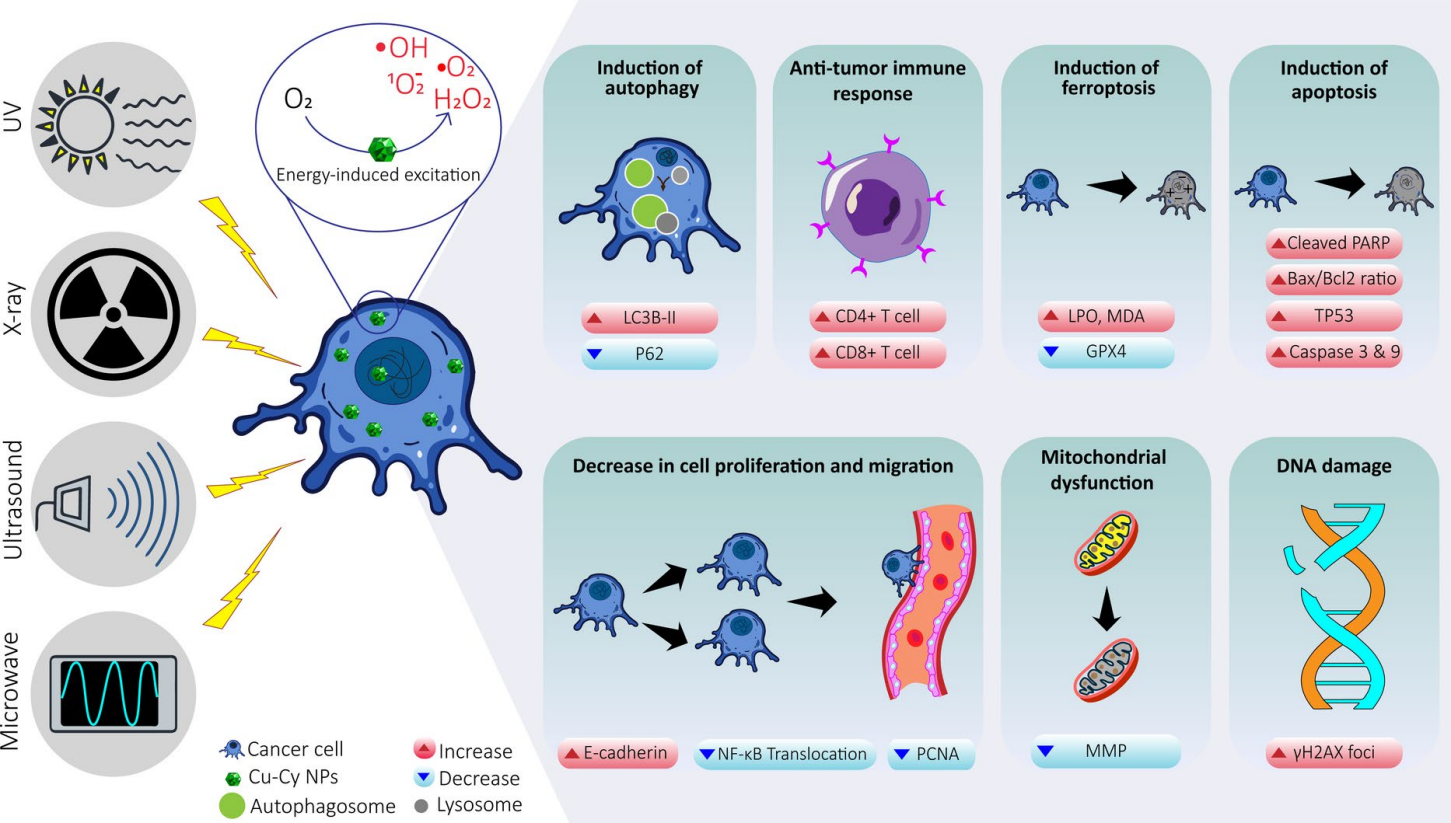

## Introduction

Despite advancements in conventional treatment modalities, such as surgery, chemotherapy, and radiation therapy, these approaches are often limited by invasiveness, systemic side effects, and non-specific tissue damage (Chen et al. 2022; Shrestha et al. 2019). Emerging therapeutic avenues, such as photodynamic therapy (PDT) and sonodynamic therapy (SDT), offer promising alternatives with a lower risk of long-term complications in cancer survivors (Shrestha et al. 2019; Wang et al. 2016).

PDT is a well-established, minimally invasive therapy that has demonstrated efficacy in managing various cancers, particularly those accessible to light, such as skin malignancies including basal cell carcinoma and actinic keratosis (Kawczyk-Krupka et al. 2020). PDT is also used clinically for several internal cancers, like esophageal, lung, and bladder cancers (Bartusik-Aebisher et al. 2022; Filonenko et al. 2016; Kimura et al. 2015). In contrast, SDT remains largely in the preclinical stage, with current research and early clinical trials exploring its potential without widespread clinical adoption (Mehta et al. 2023).

In PDT, photosensitizers mainly enter cells through passive diffusion, endocytosis, or targeted delivery when conjugated with ligands (Saenz et al. 2022). Due to the leaky tumor vasculature, photosensitizers selectively accumulate in tumor cells and vascular endothelial cells (Clement et al. 2018). Upon light exposure, these agents transfer energy to nearby triplet oxygen molecules, generating reactive oxygen species (ROS) that induce cell death (Shi et al. 2019; Wang et al. 2016).

PDT offers several advantages, including preferential accumulation of photosensitizers in tumor tissues through the enhanced permeability and retention (EPR) effect, minimal systemic toxicity, precise light targeting, the ability to treat multiple lesions simultaneously, and the option for retreatment to improve response (George et al. 2022; Huang et al. 2019b; Yu et al. 2015). While PDT can provoke DNA damage through ROS-mediated processes, this is generally less direct and less genotoxic than conventional therapies, contributing to a lower risk of secondary cancers and sustained efficacy against tumors resistant to standard treatments. Both PDT and SDT may cause side effects, including phototoxicity, oxidative tissue damage, and inflammation; however, these effects are generally milder and more localized than those associated with conventional treatments (Ma et al. 2014b; Shrestha et al. 2019).

In comparison with PDT, which uses light to activate photosensitizers, SDT employs ultrasound energy to achieve comparable effects, leading to ROS generation and subsequent cellular damage. Both PDT and SDT rely on a sensitizer that absorbs energy and transfers it to oxygen molecules, resulting in ROS production and oxidative damage to cellular structures (Gu et al. 2020; Hong et al. 2020; Sazgarnia et al. 2013; Shi et al. 2019; Wang et al. 2016).

In contrast to SDT, PDT is inherently limited by the shallow tissue penetration of light, particularly in the UV and visible ranges. Near-infrared (NIR) light allows for deeper penetration; however, it can penetrate only up to few centimetres into biological tissue, limiting PDT’s effectiveness for deep-seated tumors (Ma et al. 2014b; Mcewan et al. 2016; Shrestha et al. 2019). Moreover, PDT efficacy is reduced by rapid light attenuation as it passes through the tissues (Ma et al. 2014b; Padalkar and Pleshko 2015). Various strategies have been explored to overcome these limitations, including invasive methods such as applicators and optical fibres for enhanced light delivery (Clement et al. 2018; Shrestha et al. 2019).

Another approach, based on Förster resonance energy transfer (FRET), involves the use of transducers or photoconverting nanoparticles (NPs) that can be activated by ionizing radiation, converting X-ray energy into a usable range for photosensitizers, ultimately generating ROS and inducing oxidative stress (Shi et al. 2019). These NPs can further be combined with various photosensitizers to treat deeper tumors (Chen et al. 2019; Chuang et al. 2020). This alternative approach utilizing energy transducers to activate PDT with X-ray is known as X-ray induced photodynamic therapy (X-PDT) (Chen et al. 2022; Clement et al. 2018; Gu et al. 2020; Wang et al. 2016).

PDT can be classified into two types: Type I, where ROS are produced through electron transfer, and Type II, where ROS are generated through energy transfer from the photosensitizer to oxygen molecules (Cline et al. 2019). Microwave (MW)-triggered PDT presents another promising alternative to Type-I PDT, potentially overcoming the problems with light penetration (Zhou et al. 2023). MW exposure can induce thermal effects during the activation of sensitizers, potentially enhancing treatment effectiveness. MW radiation heats tissue, causing vasodilation and consequently boosting blood circulation. Enhanced circulation facilitates penetration of oxygen into the cancer tissue and enhances tissue necrosis and apoptosis in solid tumors, thereby augmenting the therapeutic outcome (Pandey et al. 2022; Yao et al. 2016).

Numerous photosensitizers have been developed up until today (Sun et al. 2018). First and second-generation photosensitizers including porphyrins, chlorins, phthalocyanines, phenothiazinium dyes, and natural compounds such as curcumin and hypericin, have demonstrated therapeutic potential in PDT (Chen et al. 2025; Janas et al. 2021; Machado et al. 2023; Quadrado et al. 2024; Varzandeh et al. 2021). However, many photosensitizers suffer from limitations such as poor water solubility, low tumor selectivity, photobleaching, and limited tissue penetration due to excitation wavelength restrictions (Machado et al. 2023; Wahnou et al. 2025; Wang et al. 2016). To address these shortcomings, third-generation nanophotosensitizers incorporate NPs such as gold, silica, quantum dots, carbon nanotubes, or metal oxides as carriers or enhancers; providing improved solubility, accumulation in tumoral cells, targeted delivery, surface area to volume ratio, and greater ROS generation (Bartusik-Aebisher et al. 2022; Chandak and Nagime 2025; Khanahmad et al. 2024). Among these, metal NPs are particularly well-studied due to their high stability, tunable size, surface chemistry, and multifunctionality. These NPs offer advantages such as enhanced bioavailability, controlled drug release, and the ability to serve as contrast agents in imaging or mediators in photothermal and photodynamic therapies (George et al. 2022; Shang et al. 2021).

Copper-Cysteamine nanoparticles (Cu-Cy NPs) introduced by Chen et al. in 2014 represent an emerging class of metal-based nanomaterials that combine the unique properties of copper ions with cysteamine ligands (Ma et al. 2014b). Unlike traditional metal NPs that mainly serve as carriers or contrast agents, Cu-Cy NPs act as active sensitizers with intrinsic therapeutic functions. These NPs can be stimulated by UV, but their significant advantage over other photosensitizers is their direct stimulation by X-rays, not requiring energy conversion (Ma et al. 2014b). Consequently, Cu-Cy NPs can be utilized for treating both superficial and deep-seated tumors (Ma et al. 2014b; Shrestha et al. 2019). Cu-Cy NPs can also be efficiently activated by MW and ultrasound (US) to generate potent ROS for cancer treatment (Huang et al. 2019a; Zhang et al. 2020). Therefore, they function as radiosensitizers, photosensitizers, and sonosensitizers (Chen et al. 2022; Chudal et al. 2020; Pandey et al. 2019; Shrestha et al. 2019). These NPs exhibit low cytotoxicity, cost-effective and environmentally friendly synthesis, nanoscale production for increased water solubility and cellular uptake, as well as the ability to be delivered by exosomes (Chen et al. 2019; Ma et al. 2014a; Sazgarnia et al. 2013).

This review provides the first systematic and integrative analysis of Cu-Cy NPs’ characteristics, multimodal sensitization capabilities under X-ray, UV, MW, and US exposure across various cancer types, and insights into their mechanistic roles, in both in vitro and in vivo models.

## Properties, mechanisms, and therapeutic effects of Cu-Cy NPs

### Characterization of Cu-Cy NPs

#### Photoluminescence properties:

Cu-Cy NPs with a molecular weight of 378.38 g/mol and an empirical formula of Cu₃Cl(SR)₂ (R = CH_2_CH_2_NH_2_) dispersed in deionized (DI) water exhibit a strong absorption peak at the wavelength of approximately 365 nm in UV-VIS spectroscopy, accompanied by intense orange-red photoluminescence (PL) emission when exposed to a 365 nm UV lamp or a 90kV X-rays (Ma et al. 2014b; Sah et al. 2020). This unique characteristic suggests their potential application as radiation detectors and diagnostic imaging agents in future research in addition to their ROS-generating capabilities for cancer therapy (Ma et al. 2014b, 2014a; Pandey et al. 2019).

Moreover, two additional photoluminescence emission peaks at 607 nm and 633 nm are observed upon excitation at 365 nm, corresponding to Cu(1) and Cu(2) ions, due to the transition d^9^4s^1^-d^10^ in the PLE spectra (Shrestha et al. 2019). These two types of copper ions in the NP structure act as luminescence centers, each producing distinct photoluminescence peaks due to differences in their coordination and distances to neighbouring ions. Cu(1), with shorter distances and four connections, emits at 633 nm, while Cu(2), with longer distances and three connections, emits at 607 nm, resulting in a red-orange photoluminescence spectrum with a main peak at 607 nm and a shoulder at 633 nm (Ma et al. 2014a).

#### FTIR spectrum:

The Fourier-transform infrared spectroscopy (FTIR) analysis of Cu-Cy NPs reveals characteristic peaks at 3200-3300 cm⁻^1^ and 2800 cm⁻^1^, corresponding to NH₂ and CH₂ stretching vibrations, respectively. A peak at 1600 cm^−1^ is also related to N-H bond bending, and C-C-N bending can be expected at 950 cm-1 and 1050 cm^−1^ (Akafzade et al. 2019; Chudal et al. 2020). Furthermore, C-N and C-C-N vibration peaks can be expected at 700 and 1300 cm^−1^, respectively (Chudal et al. 2020).

#### Size and physicochemical properties:

According to different protocols of Cu-Cy NPs synthesis, their diameter and size distribution may vary (Chen et al. 2022; Huang et al. 2019b; Ma et al. 2014b; Shi et al. 2019; Wang et al. 2018; Yao et al. 2016; Zhang et al. 2020). Reducing the size of the NPs can enhance their solubility and cell uptake; however, it is crucial to strike a balance between NPs size and their targeting abilities and efficacy. Generally, it is recommended that Cu-Cy NPs should be used within the size range of 50–100 nm for the best efficacy and avoiding clearance by the reticuloendothelial system (RES) (Ma et al. 2014b). Cu-Cy NPs have spherical morphology in nano-scale and make crystalline aggregates in micro-scale. Based on various scanning electron microscopy images, the morphology of Cu-Cy NPs is regular rectangular crystals (Chen et al. 2022; Chudal et al. 2020; Sah et al. 2020).

The surface coating of NPs plays a crucial role in controlling their size, enhancing colloidal stability, and improving solubility. Polyethylene glycol (PEG) is a suitable choice for this purpose (Huang et al. 2019b; Wang et al. 2018). Moreover, PEGylation, or surface coating with PEG, is highly effective in prolonging circulation time and evading uptake by the RES. It accomplishes this by reducing protein adsorption in vivo, thus aiding in RES avoidance (Ma et al. 2014b). To this end, Pandey et al. used PEG to modify the surface coating of Cu-Cy NPs and also chose an inert environment in the synthesis process, which leads to smaller sized-NPs and shorter synthesis time (Pandey et al. 2019).

The optical properties of NPs, including UV-VIS absorption, PL, and PLE peaks are related to their size and size distribution. Data suggest a direct relationship between Cu-Cy NPs size and PL intensity (Chen et al. 2022; Sah et al. 2020). This phenomenon can be attributed to the decrease in surface-to-volume ratio as NPs size grows, which is expected to amplify PL intensity. Moreover, larger NPs tend to possess better crystallinity, further augmenting PL intensity (Sah et al. 2020). Recent experiments have shown that the size distribution of Cu-Cy NPs remains consistent across various cell culture media and in different environments, in terms of acidity and alkalinity (Chen et al. 2022). Similarly, UV-VIS absorbance, PL and PLE peaks are almost the same in different physiological conditions. This consistency of physicochemical properties across various in vitro and in vivo conditions presents an advantage for pre-clinical Cu-Cy NP research and may increase their translatability and success rate in clinical trials as well.

### Anticancer effect and ROS generation mechanism of Cu-Cy NPs

Cu-Cy NPs exhibit potent anticancer effects, primarily by inducing apoptosis and inhibiting tumor proliferation in various cancer models. These NPs hold promise for cancer treatment due to their ability to act as multimodal sensitizer, generating ROS - primarily singlet oxygen and hydroxyl radicals - which cause oxidative damage to cancer cells, and promote apoptosis. Confocal fluorescence microscopy studies have shown that Cu-Cy NPs can penetrate to the cell nucleus, and their nuclear accumulation increases with longer incubation periods in cancer cells. This nuclear localization is significant because it enables ROS produced by activated Cu-Cy NPs to directly damage nuclear structures such as DNA, thereby boosting their anticancer activity (Zhang et al. 2020). Another recent study demonstrated that Cu-Cy-I NPs amplified radiation-induced DNA damage as indicated by elevated γH2AX levels, confirming their ability to penetrate the nucleus and cause double-strand breaks (Zhang et al. 2025). Furthermore, Cu-Cy NPs are also observed around mitochondria, allowing them to target multiple essential organelles for ROS-mediated cytotoxicity, and disrupt their mitochondrial membrane potential. (Liu et al. 2017). The distribution of these NPs to both the nucleus and mitochondria demonstrates superior efficacy in vitro and in vivo, with notable reduction in tumor growth and induction of cancer cell death (Chudal et al. 2020; Huang et al. 2019a; Pandey et al. 2019).

#### *ROS generation *via* photo/radio-dynamic therapy*

**Fig. 1:**
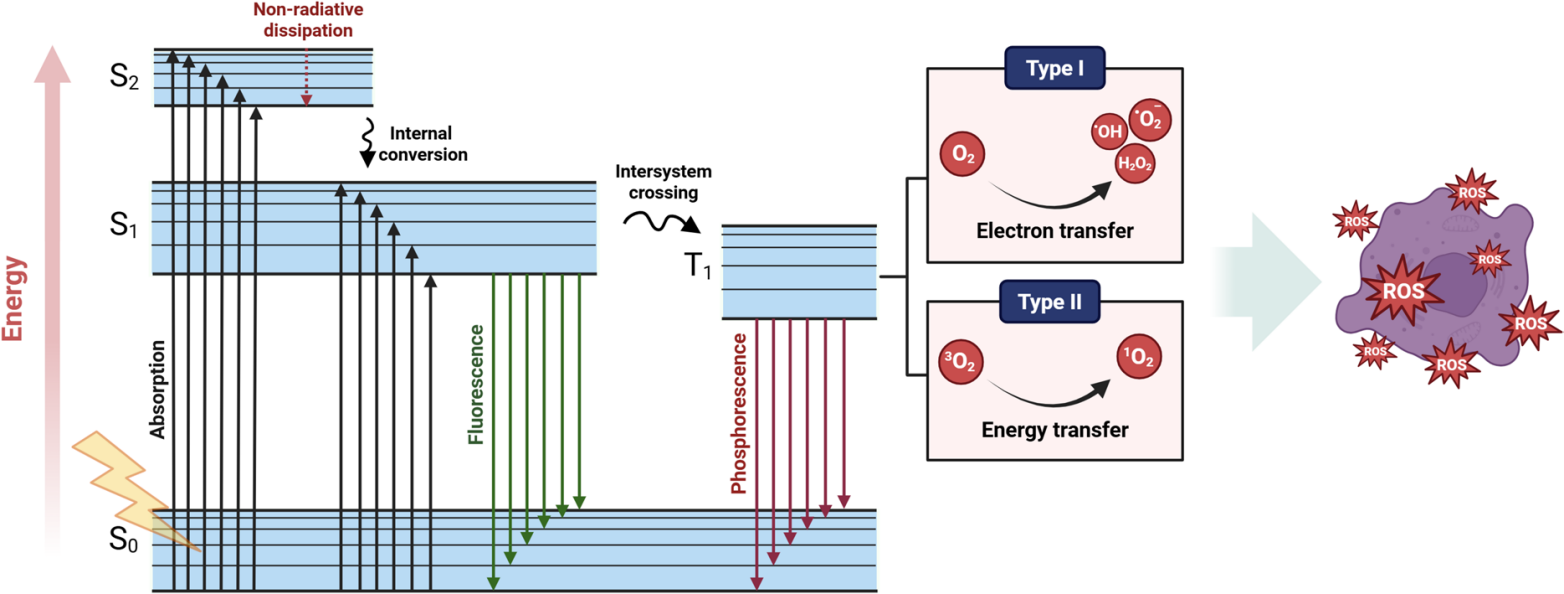
Schematic illustration of the Jablonski diagram, showing electronic transitions within a molecule. The diagram includes absorption, fluorescence, phosphorescence, and non-radiative relaxation pathways between the ground state (S_0_) and excited states (S_1_, S_2_), highlighting energy dissipation through various radiative and non-radiative processes. The Type I and Type II processes demonstrate mechanisms for ROS generation through electron transfer and energy transfer, respectively. *Modified from* (Pobłocki et al. 2021; Xu et al. 2016)

The mechanism of ROS generation by Cu-Cy NPs as photo/radio-sensitizer is illustrated in the Jablonski diagram (Figure 1) (Garcia-Diaz et al. 2016; Orsi et al. 2022). Initially, Cu-Cy NPs exist in their ground state (S_0_), characterized by electrons at low energy levels. When exposed to radiation, Cu-Cy NPs absorb the energy of photons, resulting in transition of electrons to higher energy levels (S_n_). Following absorption, some excited electrons undergo intersystem crossing (ISC) transitioning from singlet state (S_n_) to longer-lived triplet state (T_n_) (Alias et al. 2019; Wang et al. 2018). In the T_n_ state, Cu-Cy NPs can engage in two ROS generation pathways: Type I (electron transfer) and Type II (energy transfer) mechanisms. In the Type I mechanism, triplet-excited Cu-Cy NPs directly transfer electrons to molecular oxygen (O_2_), leading to the formation of superoxide radicals (^•^O_2_^-^). These superoxide radicals can undergo subsequent reactions, generating other ROS, such as hydroxyl radicals (^•^OH), or hydrogen peroxide (H_2_O_2_) (Zhen et al. 2020). In the Type II mechanism, the triplet state Cu-Cy NPs transfer energy to molecular oxygen (O_2_), promoting its transition to a highly reactive singlet oxygen (^1^O_2_) state (Orsi et al. 2022; Yang et al. 2023). ^1^O_2_ is a powerful ROS capable of inducing oxidative damage to biomolecules within cancer cells, including DNA, proteins, and lipids (Cadenas 1989; Montaner et al. 2007; Montazerabadi et al. 2012; Ramakrishnan et al. 1990). Both Type I and Type II mechanisms contribute to the cytotoxic effects of Cu-Cy NPs as photo/radio-sensitizers. The generated ROS disrupt cellular homeostasis and ultimately leading to cell death (Alias et al. 2019; Huang et al. 2019a; Montazerabadi et al. 2012; Yang et al. 2023).

The balance between Type I and Type II PDT is determined by factors such as photosensitizer properties, oxygen concentration, and substrate availability. Under hypoxic conditions, Type I pathways are generally more prominent, whereas higher oxygen levels favor Type II reactions. Since singlet oxygen is extremely short-lived and diffuses only a few nanometers, its cytotoxic action is confined to the immediate vicinity of the activated photosensitizer. Thus, the intracellular localization of the photosensitizer becomes a critical determinant of therapeutic outcome: accumulation in mitochondria often triggers intrinsic apoptosis, while localization in lysosomes or the plasma membrane tends to cause necrosis or autophagy (Gunaydin et al. 2021; Maharjan and Bhattarai 2022).

Upon exposure to UV or microwaves, Cu-Cy NPs mainly generate singlet oxygen, as evidenced by quenching studies using DABCO. They can also produce hydroxyl radicals, which have been detected via photoluminescence tracking when microwaves stimulate the NPs (Pandey et al. 2019; Wang et al. 2021; Zhou et al. 2023).

#### *ROS generation *via* SDT*

When Cu-Cy NPs are used as sonosensitizers, US stimulation induces the rapid collapse of nearby gas-filled cavities around the NPs (Wang et al. 2018). This process generates intense pressure fluctuations, releasing energy that initiates chemical reactions, including ROS production. In addition, energy of US waves can induce localized hyperthermia and mechanical damage, contributing to tumor cell necrosis through multifaceted cytotoxicity effect (Liang et al. 2020; Mcewan et al. 2016).

#### Fenton-like ROS generation and tumor-selective cytotoxicity of Cu-Cy NPs

The presence of copper ions within Cu-Cy NPs enables ROS production through a Fenton-like reaction (Chudal et al. 2020; Wang et al. 2021). This reaction involves copper ions interacting with hydrogen peroxide (H_2_O_2_) to generate hydroxyl radicals and hydroxide ions (Chang et al. 2020). The hydroxyl radicals generated through these reactions are highly reactive and can initiate chain reactions that lead to the production of additional ROS. In acidic tumor microenvironment, the rate of Fenton-like reactions increases due to the protonation of hydrogen peroxide, which enhances its reactivity with transition metals (Chen et al. 2022). As a result, oxidative stress within tumor cells is more pronounced than in normal cells (Corbet et al. 2016; LaMonte et al. 2013; Pham et al. 2013).

Notably, the presence of Cu⁺ in Cu-Cy NPs enhances the catalysis of Fenton-like reactions, as its reaction rate with H₂O₂ is approximately 22 times faster than that of Cu^2^⁺, reaching a maximum of 10^4^ M⁻^1^s⁻^1^ (Chudal et al. 2020). However, in acidic tumor microenvironments, a small amount of Cu^2^⁺ can also be released from Cu-Cy NPs, which may further enhance ROS production through classical Fenton and Haber-Weiss reactions, thereby contributing to the NPs' selective cytotoxicity in cancer cells (Chang et al. 2020; Chudal et al. 2020).

Although Cu-Cy NPs are capable of generating high levels of ROS (Wang et al. 2018; Yang et al. 2023), their specific role in regulating oxidative stress pathways across different tumor types is still under investigation. These NPs exhibit high selectivity toward cancer cells, as evidenced by lower IC_50_ values compared to normal cells. For instance, in a recent study, IC_50_ values of Cu-Cy NPs in DM6 and KYSE-30 cancer cells were approximately four times lower than those in normal HDF and HET-1A cells, underscoring their superior anticancer efficacy, while demonstrating minimal cytotoxicity to normal cells (Chudal et al. 2020).

### In vitro* studies of Cu-Cy NPs*

Cu-Cy NPs show promise as multimodal sensitizers (Chen et al. 2022; Zhang et al. 2020). These NPs have demonstrated a strong ability to enhance anti-tumor effects of X-rays across a diverse range of cancer cell lines. This includes colon, esophageal, endometrial, liver, melanoma, osteosarcoma, and breast cancer tumors, highlighting the broad applicability of Cu-Cy NPs across different cancer types (Chen et al. 2022; Liu et al. 2017; Ma et al. 2014b; Shi et al. 2019; Yang et al. 2024; Zhang et al. 2020).

Ejtema et al. (Ejtema et al. 2024) demonstrated a potent anti-tumor effect of Cu-Cy NPs driven X-PDT on human adenocarcinoma HeLa cells. The observed reduction in cell viability and migration along with increased apoptosis, was attributed to synergistic interaction between Cu-Cy NPs and X-ray irradiation. Shi et al. (Shi et al. 2019) observed a significant decrease in viability of squamous cell carcinoma (XL50) and melanoma (B16F10) cells treated with 96 nm Cu-Cy NPs-driven X-PDT, more pronounced in XL50 cells than BF1610 cells. Melanoma shows resistance to both X-ray radiation and PDT due to overexpression of tyrosinase-related protein 2 and a protective effect of melanin, which reduce ROS level and decreases light penetration. However, in the following study, optimized Cu-Cy NPs (40 nm) demonstrated significant anti-tumor effects on B16F10 cells through the X-PDT mechanism. The discrepancy in effectiveness of 40 nm and 96 nm Cu-Cy NPs-driven X-PDT was explained by increase of surface-to-volume ratio in smaller NPs that led to generation of higher concentration of ROS upon stimulation. Furthermore, reduction of NP size, resulted in increased cellular uptake (Ma et al. 2014b; Sah et al. 2020).

Liu et al. (Liu et al. 2017) suggested that the efficacy of Cu-Cy NPs-driven X-PDT depends on both particle concentration and X-ray dose. While low doses showed minimal effect, higher doses (2–3 Gy) significantly increased X-PDT efficacy. However, at 4 Gy, efficacy plateaued and even slightly decreased, indicating non-linear dose-dependence. Thus, the optimal outcome is not necessarily achieved by increase of radiation dose, but efficacy of the treatment is rather influenced by interaction of X-rays and Cu-Cy NPs resulting in production of ROS and death of cancer cells. Additionally, Cu-Cy activation may lower mitochondrial membrane potential. Taken together, these findings may reflect target depletion for ROS generation or ionization. The reduced efficacy at 4 Gy compared to 3 Gy may result from saturation of ROS production, activation of cellular protective mechanisms at higher doses, cellular stress responses or repair mechanisms, aligning with radiobiological phenomena, where excessive doses diminish effectiveness.

**Fig. 2:**
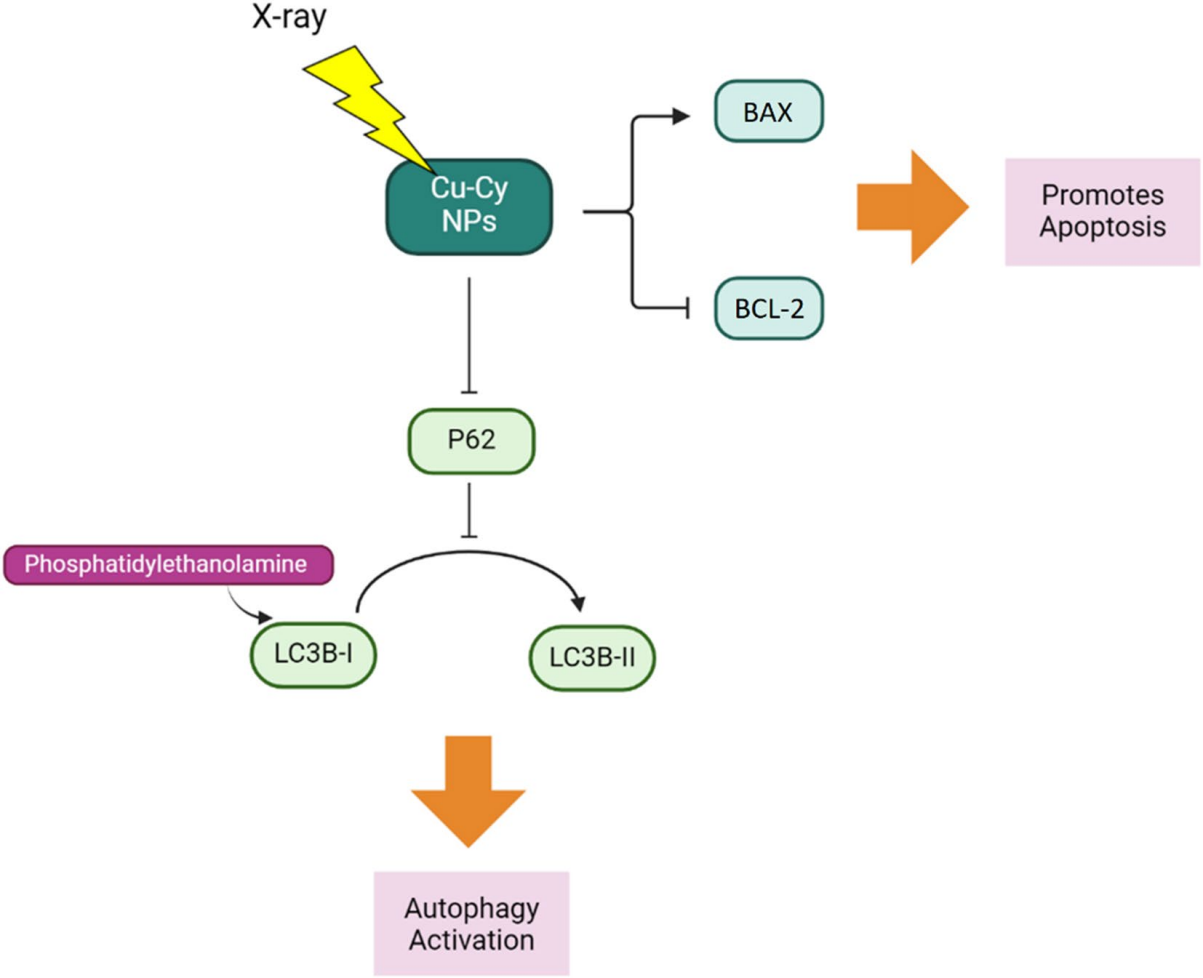
Molecular pathways of X-ray activated Cu-Cy NPs effects on SW620 cells

While work is still ongoing to fully elucidate the exact mechanisms by which Cu-Cy NPs exert their anti-tumor effects, several key pathways have been identified. X-ray activation of Cu-Cy NPs increases BAX expression, while decreasing BCL-2 expression (Liu et al. 2017), resulting in a shift in the BAX/BCL-2 ratio that activates the apoptosis. Furthermore, a significant decrease in P62, a protein involved in autophagosome formation, suggesting activation of autophagy. Indeed, conversion of LC3B-I to LC3B-II, a marker of autophagic activity, is notably increased after treatment with X-ray activated Cu-Cy NPs (Figure 2). Consistent with these findings, transmission electron microscopy (TEM) revealed the presence of numerous double-membrane autophagic vacuoles containing cytoplasmic components and organelles in SW620 cells treated with X-ray activated Cu-Cy NPs, further confirming the induction of autophagy (Liu et al. 2017).

Building on the findings with X-ray irradiation, Liu et al. (Liu et al. 2025) further evaluated the effects of gamma-ray exposure using a Gamma Knife system. Upon gamma-ray activation, Cu-Cy induced a significant, dose-dependent decrease in cancer cell viability, with HuH-7 cells being more sensitive than HepG2 and normal cells. Cu-Cy combined with gamma irradiation also significantly inhibited cancer cell migration in a concentration-dependent manner. Mechanistically, ROS production was markedly increased in Cu-Cy-treated cells post gamma irradiation, highlighting ROS-mediated oxidative stress as the cause of cell death and migration inhibition.

**Fig. 3:**
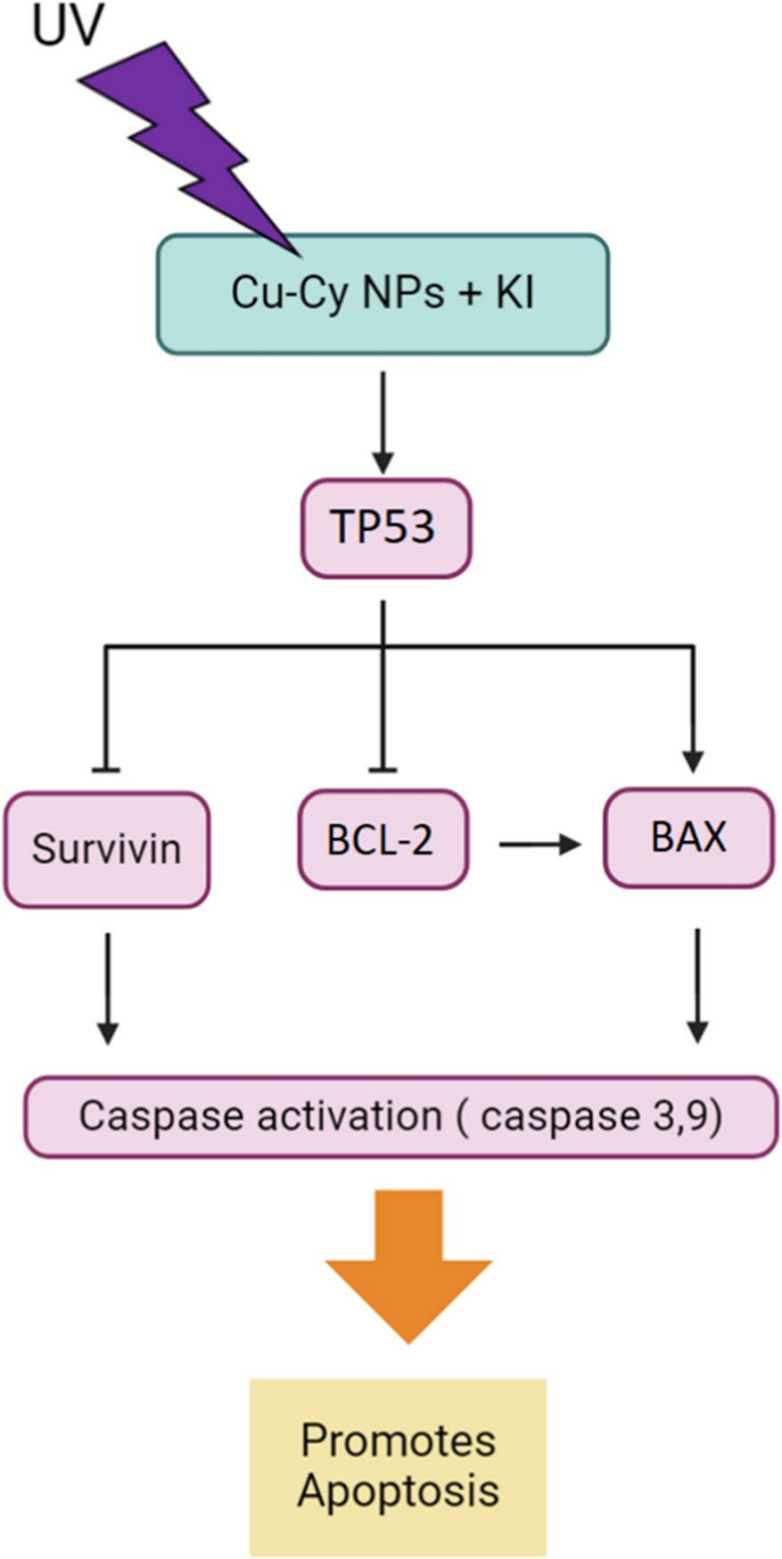
Cu-Cy NPs combined with potassium iodide (KI) under UV exposure enhance apoptosis in liver cancer cells

Cu-Cy NPs also demonstrate a considerable ability to work synergistically with other drugs. Chang et al. (Chang et al. 2020), reported that Cu-Cy NPs were able to enhance anti-tumor effect of Disulfiram (tetraethylthiuram disulfide, DSF) in esophageal cancer cells. This synergistic effect was most pronounced when the DSF and Cu-Cy NPs were combined at a molar ratio of 1 to 4, resulting in a DSF to Cu^2+^ ratio of 1 to 12. This combination worked by inducing ROS accumulation and suppressing the NF-κB signaling, a pathway frequently constitutively activated in malignant cells that promotes pro-inflammatory cytokine expression and facilitates tumor progression and metastasis (Habeeb & Sugumaran 2022; Wahnou et al. 2025). Similarly, (Yang et al. 2024) demonstrated that DSF combined with Cu-Cy NPs induced mitochondrial damage and apoptosis in endometrial cancer cells. The combination impaired mitochondrial function, disrupted the DNA repair pathway, and promoted apoptosis. In addition, Zhen et al. (Zhen et al. 2022) found that Cu-Cy NPs in combination with potassium iodide (KI) under UV exposure effectively suppressed the growth, migration, and colony formation of these cancer cells. Cu-Cy NPs combined with KI for PDT synergistically induced apoptosis and disrupted mitochondrial function. This combination increased ROS level while decreasing mitochondrial membrane potential (MMP). Moreover, Cu-Cy NPs-mediated PDT elevated TP53 expression in both HepG2 cells and tissues, reduced survivin expression and the BCL-2/BAX ratio, and activated caspase-3 and caspase-9 (Figure 3).

Recent research by Zhang et al. (Zhang et al. 2025) has further expanded the therapeutic potential of Cu-Cy NPs by developing iodinated Cu-Cy NPs (Cu-Cy-I NPs) as efficient radiosensitizers for tumor radiotherapy. Upon X-ray irradiation, these NPs significantly enhanced ROS production in 4T1 breast cancer cells, leading to increased DNA damage, as evidenced by elevated γH2AX foci formation.

Cu-Cy NPs can also be activated by UV radiation. Huang et al. (Huang et al. 2019b) reported that low concentrations of Cu-Cy NPs effectively reduced cancer cell viability when followed by UV irradiation. Notably, Cu-Cy NPs could enter exosomes secreted by the tumor cells, facilitating targeted delivery to other cancer cells. In addition, UV-activated Cu-Cy NPs markedly increased apoptosis, as evidenced by enhanced expression of cleaved-PARP.

Zhou et al. (Zhou et al. 2023) evaluated the effectiveness of Cu-Cy NPs-mediated MW dynamic therapy (MWDT) on colorectal cancer cells. The exposure of the Cu-Cy NPs to MW radiation resulted in increased cytotoxicity in NPs a concentration dependent manner. Cu-Cy NPs-mediated MWDT enhanced lipid peroxides (LPO) and malondialdehyde (MDA) levels, potentially indicating ferroptosis induction. The hypothesis was further supported by reduction of glutathione peroxide 4 (GPX4) expression in treated cells. In a similar study on osteosarcoma cells, Yao et al. (Yao et al. 2016) reported a higher level of ROS production and apoptosis in the Cu-Cy NPs driven MWDT treated cells; however, the main mechanism underlying the anti-tumor effect was identified as heat generation and the release of copper ions from Cu-Cy NPs. Nonetheless, the cell-killing effect was not directly heat-induced, as hyperthermia typically causes cell rounding due to cytoskeletal condensation, which was not observed here. This suggests that hyperthermia probably contributed to ROS production, amplifying the cytotoxic effect.

Cu-Cy NPs have also been evaluated for their efficacy as sonosensitizers for SDT in breast cancer treatment (Wang et al. 2018). SDT is a non-invasive, high safety method with ability to achieve penetration up to 15 cm. Cytotoxicity of breast cancer cells treated with Cu-Cy NPs markedly increased upon US irradiation. Furthermore, there was a notable increase in intracellular ROS level, apoptotic rate, and cell membrane damage.

Yang et al. (Yang et al. 2023) developed a heterogeneous nanoplatform (NSCuCy) by assembling aggregation-induced emission luminogens active type-I photosensitizers (NS-STPA) and Cu-Cy NPs for PTD. In vitro studies using MCF-7 cells demonstrated that NSCuCy significantly increased intracellular ROS production under 660 nm laser irradiation, as compared to NS-STPA or Cu-Cy NPs alone. Moreover, the nanoplatform exhibited minimal cytotoxicity in the dark, especially for normal (MCF-10A) cells. NSCuCy enhanced generation of •OH, mainly through type-I PDT mechanism.

Table 1 summarizes the in vitro studies included in this review, which investigated the effect of Cu-Cy NPs on a variety of cancer cell lines using various irradiation sources and conjugation strategies.

**Table 1:** In vitro studies on the application of Cu-Cy NPs under various stimulations

Stimuli	Cell line	Cu-Cy NPs size [nm]	Cu-Cy NPs concentration	Pre-stimulation incubation time/Stimulation source properties	Main findings	Reference
X-ray	HepG2, SK-HEP-1 Li-7, 4T1	43 ± 10	0–150 mg/L (50, 100 mg/L*)	[6h, 24 h] 2 Gy (6 MV, 100 MU/min)	↓ Proliferation, migration, PCNA ↑ ROS, E-cadherin	(Chen et al. 2022)
	XL50, HaCaT, B16F10	95.7 ± 8.4	0.1–200 µg/mL	[2h] 1.5 Gy (100 kVp)	↓ Viability - B16F10 cells are relatively resistant to X-PDT	(Shi et al. 2019)
	B16F10	~ 40	0–100 µg/mL (100 µg/mL*)	[24h] 2.5 Gy	↑ Apoptosis and/or necrosis, cellular uptake, oxidative stress, anti-tumor immune response ↓Cell viability	(Zhang et al. 2020)
	MCF-7	250	0–200 μg/mL (40 μg/mL*)	[24h] 2 Gy	↑ ROS ↓ Cell viability	(Ma et al. 2014b)
	SW620	NA	0–100 mg/L (50, 100 mg/L*)	[4h] 0, 1, 2, 3, 4 Gy (90 kV, 5 mA, 0.5 Gy/min)	↓Viability, MMP^1^, P62, BCL-2 ↑Apoptosis/autophagy, BAX conversion of LC3B-I to LC3B-II, Autophagic vacuole formation	(Liu et al. 2017)
	HeLa	46 ± 18	0–100 mg/L (25mg/L*)	[24h] 0, 2, 4 Gy (6 MV, 200 cGy/min)	↓ Viability, Migration ↑ Apoptosis	(Ejtema et al. 2024)
KI + X-ray	4T1, ID8, OVCAR3, A2780	800	(0–100 μg/mL) (10 μg/mL*)	[4h, 6h, ~12h] 2, 4, 6 Gy (1.212 Gy/min)	↑ROS, γH2AX foci, DNA damage and cytotoxicity	(Zhang et al. 2025)
UV	HepG2	~ 100	0–200 µg/mL (25 µg/mL*)	[Immediately] 6 J/cm^2^ (irradiation peak at 360 nm, 20 mW/cm^2^, 0–10 min),	↓ Cell viability ↑ Apoptosis ↑Cleaved-PARP - Exosome-mediated delivery	(Huang et al. 2019b)
KI + UV	HepG2 Hep3B Huh7	NA	0–50 μg/mL (12.5 μg/mL*)	[NA] 6 J/cm2 0–10 min	↓ Growth, Migration, Colony formation, MMP, BCL-2/BAX, Survivin ↑ Apoptosis, ROS, TP53, Caspase-3, Caspase-9	(Zhen et al. 2022)
MW	HCT15	NA	0–80 μg/mL (20 μg/mL*)	[24h] 2450 MHz, 20 W, 3min	↑ Cytotoxicity, Ferroptosis, LPO, MDA ↓GPX4	(Zhou et al. 2023)
	UMR 106–01	250	0, 6.25, 25 μg/mL	[24h] 2450 MHz, 20 W, 5min	↑ Cell apoptosis, Level of ROS, ↓ Cell Viability	(Yao et al. 2016)
US	MCF-7, 4T1, MDA-MB-231	10	0.5, 1, 2 μM	[24h] 0.5 W.cm^−2^ duty cycle: 20%, duration: 60s	↑ ROS, Apoptosis/necrosis, Membrane damage	(Wang et al. 2018)
DSF	KYSE-30	NA	Cu-Cy: 1 μM, 4 μM, 8 μM DSF: 1 μM, 10 μM, 50 μM (4 μM Cu-Cy, 1 μM DSF*)	[simultaneously] NA	↑ ROS ↓ Proliferation NF-ƙB (p65) translocation	(Chang et al. 2020)
	Ishikawa, HUVEC	NA	0.5 μM Cu-Cy + 0.5 μM DSF	[simultaneously] 2463 nm (Z-Average size)	↓Angiogenesis, Colony formation, MMP ↑Apoptosis	(Yang et al. 2024)
Laser	MCF-7 MCF-10A	120	0, 5.5, 11, 28, 56*, 84 μg/mL (NSCuCy)	[16h] 660 nm, 0.4 W/cm^2^, 5min	↑Cell mortality, Apoptosis, ^•^OH generation	(Yang et al. 2023)
Gamma-ray	HepG2, HuH-7, L02	2288, 1851	0–100 μg/mL (25, 50 μg/mL*)	[24h] 2, 4 Gy	↑ ROS ↓ Proliferation, Migration	(Liu et al. 2025)

^*^If concentration range is reported, asterisk (*) indicates the concentration used in the majority of in vitro experiments within that study

### In vivo studies and preclinical efficacy of Cu-Cy NPs

Cu-Cy NPs have been used for tumor eradication in several in vivo studies. While Cu-Cy NPs alone show minimal antitumor effect (Zhen et al. 2022), they demonstrate significant therapeutic potential when stimulated with different forms of energy. Table 2 summarizes in vivo pre-clinical studies done so far.

**Table 2:** In vivo Studies and Preclinical Efficacy of Cu-Cy NPs under various stimulations

Stimuli	Animal	Tumor model (Cell type/Cancer type)/ Cell injection site	Tumor location and primary tumor size	Cu-Cy NPs concentration and injection type	Cu-Cy NPs size [nm]	[Post-injection time before stimulation]/Stimulation source properties	Review time (days)	Main findings	Reference
X-ray	Nude mice	MCF-7 cells (Human breast adenocarcinoma), Inoculated subcutaneously	Shoulder and leg (4mm)	0.8 mg/mL (10 µL-right shoulder, 15 µL-left leg, 20 µL-right leg), Intratumorally	250	[30 min] 5 Gy	12	Tumor volume significantly reduced	(Ma et al. 2014b)
	BALB/c mice	JC cells (Murine mammary adenocarcinoma), Inoculated subcutaneously	NA (5-8mm)	0.8 µg/µL (20 µL solution containing 16 µg of Cu-Cy NPs), Intratumorally	40, 100, 300 (Conjugated to pHLIP)	[30min] 5 Gy, (90, 250 and 350 kVp)	28	Smaller NP sizes (~ 40 nm) and lower radiation energies were most effective in reducing tumor volumes	(Sah et al. 2020)
	Female BALB/c nude mice	4T1 cells (Murine mammary carcinoma), Inoculated subcutaneously	Back (5mm)	50 μL (1 μg/μL), Intratumorally	43 ± 10	[30min] 2 Gy, (6 MV, 100 MU/min)	10	Tumor growth inhibition and no significant toxicity in both mice and rabbits	(Chen et al. 2022)
	New Zealand white rabbits	VX2 tumor fragments (Rabbit squamous cell carcinoma), Inoculated in situ	Liver (NA)	3 mL (1 mg/mL), Intratumorally		[30min] 2 Gy, 6 MV, 100 MU/min	7		
	SKH-1 hairless mice	XL50 cells (Murine squamous cell carcinoma) mixed with 3T3 fibroblast cells (Murine fibroblasts) (ratio: 5:1), Inoculated subcutaneously	Back (200 mm^3^)	0.1 mL (0.8 mg/mL), Multipoint intratumorally	95.7 ± 8.4	[3h] 2 Gy 100 kVp	16	Tumor growth inhibition, reduction in microvascular density, safety in mice	(Shi et al. 2019)
	C57BL/6 mice	B16F10 cells (Murine melanoma), Inoculated subcutaneously		0.1 mL (0.8 mg/mL), Multipoint intratumorally		[3h] 2 Gy, 4Gy 100 kVp	10	Only temporarily inhibited the growth, Safety in mice	
	BALB/c mice	CRL-2116 cells (JC Breast murine cancer), Inoculated subcutaneously	Right flank (4-8mm)	20 μL (0.8 μg/μL), Intratumorally	∼ 200 (Conjugated / Not Conjugated to pHLP)	[30min] 5 Gy, 90 kVp, 30 mA	57- 93	Enhanced tumor growth inhibition	(Shrestha et al. 2019)
DSF	Female BALB/c nude mice	Ishikawa cells (Human endometrial adenocarcinoma), Inoculated subcutaneously	Back (50 mm^3^)	30 μL (CuCy (0.5 μM) and DSF (0.5 μM))	2463 nm (Z-Average size)	[simultaneously] NA	12	Enhanced anti-tumor efficacy, inhibition of angiogenesis	(Yang et al. 2024)
	Male NCr nu/nu nude Mice	KYSE-30 cells (Human esophageal squamous cell carcinoma), Inoculated subcutaneously	Back (5 mm)	Cu-Cy (4 µM) + DSF (1 µM) (amounts were according to the body weight of that day)	NA	[simultaneously] NA	32	Tumor growth inhibition, no noticeable histological toxicity	(Chang et al. 2020)
MW	Female athymic BALB/c nude mice	HCT15 cells (Human colorectal adenocarcinoma), Inoculated subcutaneously	Left flank (5–8 mm)	30μL (1 μg/μL), Injections on 0, 3rd, 6th and 9th day)	NA	[1st, 4st, 7st, and 10th day] Directly to the tumors through a radiator probe	10	Tumor growth inhibition	(Zhou et al. 2023)
	Female nude C57BL/6 mice	UMR-106 cells (Rat osteosarcoma), Inoculated subcutaneously	Both the shoulder And the leg (5–8 mm)	30, 50, 100 µL (1 mg/mL), Intratumorally	250	[30 min] 2450 MHz, 20 W, 5min	14	Tumor necrosis, good biocompatibility, low toxicity	(Yao et al. 2016)
UV	Nude mice	HepG2 cells (Human hepatocellular carcinoma), Inoculated subcutaneously	Shoulder and flank (5 mm)	1 mg/mL, 100 μl/day, Three consecutive days	100	[NA] 5min 6 J/cm^2^	30	Tumor growth inhibition	(Huang et al. 2019b)
US	Female, BALB/c mice	4T1 cells (Murine mammary carcinoma), Inoculated subcutaneously	Right flanks (40–80 mm^3^)	0.25, 0.5, and 0.75 mg kg^−1^ (every 2 days, 4times). intratumorally	10	[30min] 2 W, 1.0 MHz, 3 min	13	Decrease in tumor size, significant levels of ROS production in tumor, no considerable adverse effects	(Wang et al. 2018)
Laser	BALB/c nude mice	MCF-7 cells (Human breast adenocarcinoma), Inoculated subcutaneously	Right flanks (100 mm^3^)	50 µL, (1.0 mg/mL), Intratumoral injection	120 (NSCuCy platform)	[16h] 660 nm 0.4 W/cm^2^ 5 min	14	Enhance tumor destruction, High biocompatibility and low toxicity of NSCuCy	(Yang et al. 2023)
KI + UV	Male BALB/c nude mice	HepG2 cells (Human hepatocellular carcinoma), Inoculated subcutaneously	Right upper forelimb (5–6 mm)	12.5 μg/mL and a mixed solution of 3.75 mg/mL with KI, Intratumoral injection (Once a day for 3 days)	NA	[NA] 6 J/cm2 5 min (each day for the first three days)	30	Tumor inhibition, safe and effective treatment	(Zhen et al. 2022)
KI + X-ray	BALB/c mice	4T1 cells (Murine mammary carcinoma), Inoculated in situ	Fourth mammary gland on the left side (200–300 mm^3^,)	40 µg/100 µL tumor volume (Cu-Cy-I NPs in PBS: 0.8 mg/mL), Intratumoral injection	800	[30min- for first irradiaton], 5 Gy every 3 days (20Gy in total), 4.5 Gy/min	40	Enhanced tumor suppression, extended survival in mice	(Zhang et al. 2025)
[^131^I]NaI				40µg/100 µL tumor volume [^131^I]Cu-Cy-I and additive 50 µCi of ^131^I/100 µL tumor volume, Intratumoral injection		__		High radiolabeling stability in vivo, slightly delayed tumor growth, but did not significantly prolong survival	
Gamma-ray	SPF female BALB/c mice	H22 cells (Murine hepatocellular carcinoma), Inoculated subcutaneously	(4–5 mm)	50 µL (1 mg/mL), Intratumoral injection	2288, 1851	[30min] 2, 4 Gy	14	Slowed tumor growth, increased necrosis, increased E-cadherin, reduced proliferation marker Ki-67, no major toxicity observed	(Liu et al. 2025)

In general, all studies revealed a synergistic effect between Cu-Cy NPs and various external stimuli, leading to enhanced tumor reduction. For instance, Cu-Cy NPs conjugated with KI and exposed to UV light exhibited superior tumor growth inhibition compared to Cu-Cy NPs alone (Zhen et al. 2022). Additionally, Cu-Cy NPs in combination with DSF (Chang et al. 2020; Yang et al. 2024), or under UV (Huang et al. 2019b; Zhen et al. 2022), X-rays (Chen et al. 2022; Ma et al. 2014b; Sah et al. 2020; Shi et al. 2019; Shrestha et al. 2019), MW (Yao et al. 2016; Zhou et al. 2023), laser (Yang et al. 2023), and US (Wang et al. 2018) stimulation have also been reported to be effective in cancer treatment.

In vivo studies on Cu-Cy NPs have shown significant potential for hepatocellular carcinoma (HCC) treatment. Huang et al. (Huang et al. 2019b) demonstrated that Cu-Cy NPs under UV light inhibited growth of subcutaneous HepG2 cells xenografts in nude mice, with apoptosis as a primary mechanism of cell death. Continuing studies on UV as a stimuli on a similar model, Zhen et al. (Zhen et al. 2022) combined Cu-Cy NPs with KI, which improved tumor inhibition in vivo. The outcome achieved a level comparable to the established chemotherapy drug 5-FU. These studies suggest that Cu-Cy NPs, particularly when combined with KI, are a very promising approach for enhancing PDT in HCC treatment. In another study on HCC conducted by Liu et al.(Liu et al. 2025), the combination of Cu-Cy injection and gamma knife irradiation substantially suppressed tumor growth. Tumors treated with combination therapy exhibited marked necrosis, decreased proliferation (Ki-67), and increased E-cadherin expression, indicating reduced tumor invasiveness. No significant toxicity or weight loss was observed in treated mice, and histopathology of major organs showed no abnormality, suggesting good biocompatibility.

Zhang et al. (Zhang et al. 2025) further evaluated the therapeutic efficacy of Cu-Cy-I NPs in an orthotopic 4T1 breast cancer mouse model. Treatment with Cu-Cy-I NPs combined with X-ray irradiation effectively suppressed tumor growth and prolonged animal survival without notable systemic toxicity. These results highlight the promise of Cu-Cy-I NPs as effective radiosensitizers, offering a new strategy for enhancing the efficacy of radiotherapy in cancer treatment. Additionally, the study demonstrated that ^131^I-labeled Cu-Cy-I NPs exhibited high radiolabeling stability in vivo, but at the tested low dose, their local therapeutic effect was limited, as they primarily remained at the injection site and did not significantly improve tumor control or survival compared to the combination of Cu-Cy-I NPs with X-ray irradiation.

The effects of Cu-Cy NPs on tumor growth vary depending on factors, such as dosage, NP size and conjugation strategies, as well as energy of the applied radiation (Chen et al. 2022; Ma et al. 2014b; Sah et al. 2020; Shi et al. 2019; Shrestha et al. 2019). Ma et al. (Ma et al. 2014b) reported that while Cu-Cy NPs alone showed minimal toxicity and did not significantly reduce tumor size, higher doses of Cu-Cy (20 µL of 0.8 mg/mL suspension) activated by X-ray markedly decreased tumor volume, emphasizing the necessity of X-ray activation for efficacy. Chen et al. (Chen et al. 2022) described the use of Cu-Cy NPs combined with X-PDT in breast and liver tumor models. The results indicated that Cu-Cy NPs showed promising potential in reducing tumor growth when combined with X-ray irradiation, without causing significant toxicity in mice and rabbits. Histological evaluation revealed necrosis in tumor tissues treated with Cu-Cy-based X-PDT, suggesting its effectiveness in inhibiting cell proliferation and migration via modulation of key biomarkers like E-cadherin and PCNA. Similarly, Shi et al. (Shi et al. 2019) evaluated Cu-Cy-based X-PDT in mouse xenografts model of squamous cell carcinoma (SCC) and melanoma. The results demonstrated that X-PDT effectively reduced microvessel density in SCC tissues and inhibited tumor vascularization, though the treatment was not effective in the melanoma model. Histopathological analysis showed significant reductions in tumor vessels in the X-PDT group compared to the control, especially in squamous cell carcinoma tissue.

Shrestha et al. (Shrestha et al. 2019) and Sah et al. (Sah et al. 2020) took a different approach by conjugating Cu-Cy NPs with pH-low insertion peptide (pHLIP) to enhance tumor targeting. Shrestha et al. (Shrestha et al. 2019) found that plain Cu-Cy NPs, when used with X-ray radiotherapy, did not result in a significant tumor size reduction compared to X-rays alone. However, when Cu-Cy NPs were conjugated with pHLIP, a significant reduction in tumor size was observed compared to both radiation-only treatment and radiation combined with plain Cu-Cy NPs. These findings indicate that pHLIP-conjugated Cu-Cy NPs are more effective in reducing tumor size under X-ray activation than pristine Cu-Cy NPs, highlighting the need for further research to optimize the use of NP conjugation in combination therapies. Sah et al. (Sah et al. 2020) also utilized pHLIP-conjugated Cu-Cy NPs, but focused on optimizing NP size and radiation energy to maximize therapeutic outcomes, finding that smaller Cu-Cy NPs (40 nm) were the most effective in reducing tumor size upon X-ray irradiation, while larger NPs (300 nm) had the weakest effect. Interestingly, mid-sized NPs (100 nm) produced the highest ROS, but were not the most effective at tumor suppression. Additionally, lower radiation energy (90 kVp) resulted in better tumor reduction than higher energies, underscoring the complexity of optimizing these parameters for effective treatment.

Yang et al. (Yang et al. 2023) constructed a heterogeneous nanoplatform (NSCuCy) by assembling aggregation-induced emission luminogens active type-I photosensitizers (NS-STPA) and Cu-Cy NPs and demonstrated that NSCuCy is highly effective for tumor ablation via type-I PDT. In vivo studies on MCF-7 tumor bearing mice showed selective accumulation of NSCuCy at tumor sites and significant tumor growth inhibition, when combined with light exposure. The NSCuCy worked by generating high levels of hydroxyl radicals (•OH), resulting in nearly total tumor ablation. Histological analysis confirmed extensive tumor damage, while no adverse effects, such as weight loss or organ damage, were observed, indicating good biocompatibility of NSCuCy.

Yo et al. (Yao et al. 2016) and Zhou et al. (Zhou et al. 2023) both demonstrated that Cu-Cy NPs, when activated by MW radiation, significantly reduced tumor growth. Yo et al. (Yao et al. 2016) showed that higher doses of Cu-Cy NPs combined with MW led to tumor necrosis, while the NPs alone or MW alone had little effect. Upon MW activation, Cu-Cy NPs-treated tumors exhibited signs of necrosis, such as nuclear pyknosis and cytoplasmic edema. Biodistribution studies revealed that Cu-Cy NPs predominantly concentrated in the lungs and liver, where they were eventually cleared, indicating low toxicity and good biocompatibility.

Wang et al. (Wang et al. 2018) demonstrated that Cu-Cy NPs, when activated by US, generated significant levels of ROS in tumors, leading to cancer cell destruction. In contrast, Cu-Cy NPs alone or US alone did not produce noticeable levels of ROS. Tumor cell apoptosis was induced with higher Cu-Cy NPs doses, while no significant side effects were noticed, as indicated by stable body weight in treated animals.

Chang et al. (Chang et al. 2020) and Yang et al. (Yang et al. 2024) evaluated anti-tumor efficacy of DSF combined with Cu-Cy NPs in vivo, particularly in xenograft mouse models of esophageal cancer and endometrial cancer. DSF is an FDA-approved drug that has demonstrated antitumor activity, particularly when combined with copper (Yang et al. 2024). Chang et al. demonstrated that the combination of DSF and Cu-Cy NPs significantly reduced tumor growth in KYSE-30 xenograft mice. Importantly, the combined treatment did not result in noticeable histological changes, indicating the safety of the dual-therapy approach. However, in the groups treated with DSF + CuCl₂, signs of tissue damage were observed in the heart, kidney, spleen, and liver. Moreover, single-agent treatment (either DSF or Cu-Cy NPs alone) had little to no impact on tumor growth. Yang et al. also reported potent anti-tumor effects of Cu-Cy NPs combined with DSF, while also affecting angiogenesis and dismantling already formed blood vessels. Moreover, mice treated with DSF alone exhibited weight loss and impairment of kidney function, as evidenced by elevated serum creatinine and uric acid levels. In contrast, mice receiving the combined treatment displayed fewer side effects. Liver function indicators (ALT, AST, γ-GT) remained normal across all groups, while kidney function parameters showed mild disturbances primarily in the DSF-only group. These findings suggest that the addition of Cu-Cy NPs can enhance the anti-cancer effect of DSF and alleviate its systemic toxicity in vivo. Although very low doses of DSF were used in these studies, its intrinsic toxicity and dependence on Cu^2^⁺ for full antitumor activity should be carefully considered in clinical translation.

In addition to their antitumor efficacy, Cu–Cy-based PDT activated a robust antitumor immune response in vivo. Treatment with Cu–Cy NPs and X-ray irradiation significantly increased the levels of CD4⁺ and CD8⁺ T cells in the spleen, while having minimal effects on other systemic immune cells. In the tumor microenvironment, flow cytometry revealed increased infiltration of dendritic cells (DCs), CD8⁺ T cells, and NK cells. This immune activation was accompanied by reduced levels of immunosuppressive M2 macrophages. These findings suggest that Cu–Cy-mediated PDT not only inhibits tumor growth directly but also modulates the tumor immune microenvironment by promoting DC maturation (Zhang et al. 2020).

## Literature search strategy

**Fig. 4:**
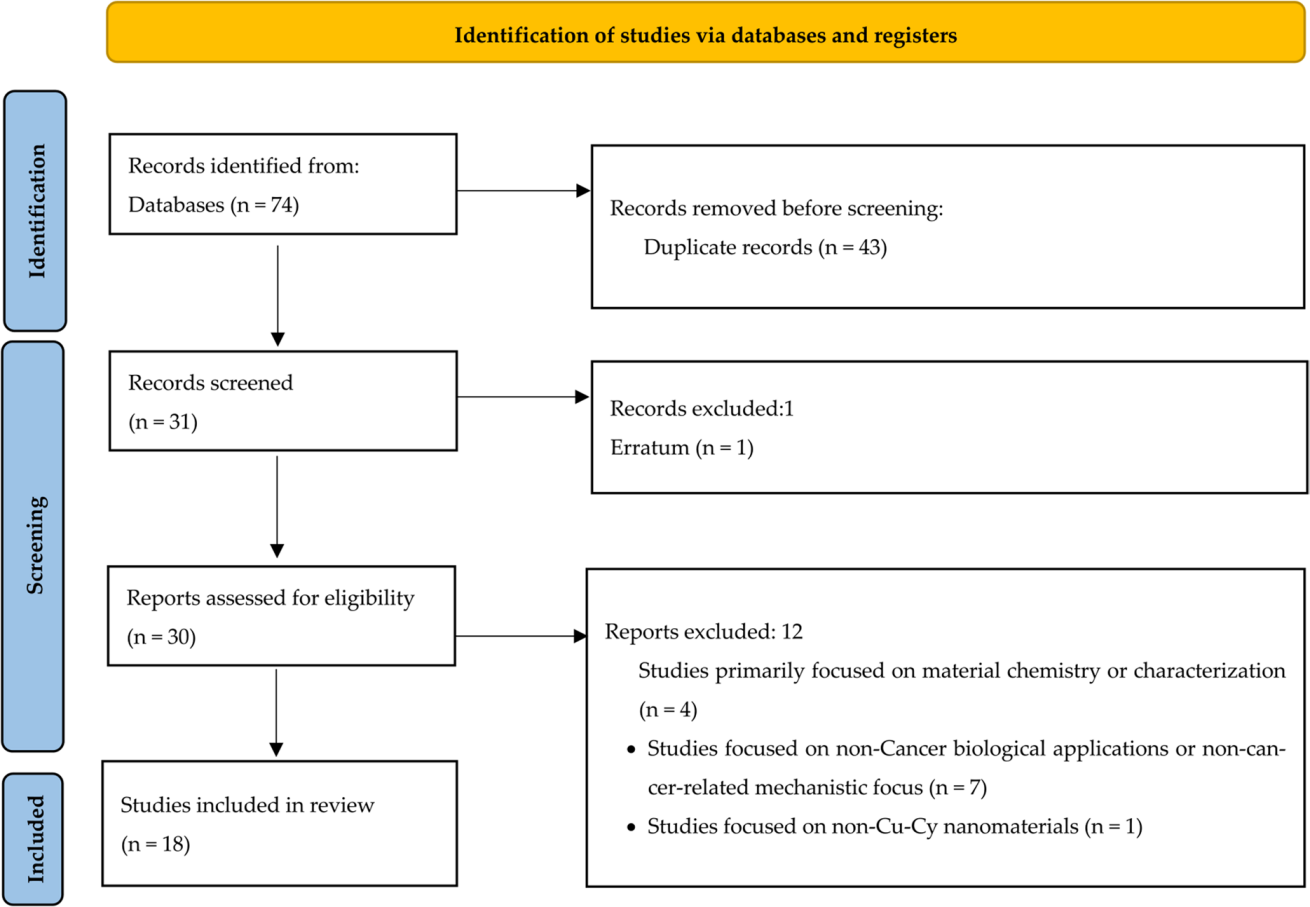
PRISMA flow diagram outlining the study selection process for the systematic review on Cu-Cy NPs in cancer treatment, covering articles published up to August 2025. The diagram depicts the stages of identification, screening, eligibility, and inclusion, detailing the number of records retrieved from PubMed, Scopus, and Web of Science, as well as records excluded due to duplication, irrelevance, or exclusion criteria (e.g., reviews, book chapters, errata, short surveys, and conference papers)

This systematic review follows the Preferred Reporting Items for Systematic reviews and Meta-Analyses (PRISMA) guidelines. We searched three major indexing databases: PubMed, Scopus, and Web of Science. The search covered articles published until August 2025 and was restricted to English language publications. The search strategy utilized a combination of keywords to identify relevant articles. Boolean operators (AND, OR) were used to combine search terms. Articles were defined using the following search strings: (Copper-Cysteamine nanoparticles AND Cancer) OR (Copper-Cysteamine nanoparticles AND (Photosensitizer OR Photodynamic therapy OR X-PDT OR X-ray induced)) OR (Copper-Cysteamine nanoparticles AND (Radiosensitizer OR Radiation)) OR (Copper-Cysteamine nanoparticles AND (Sonosensitizer OR Sonodynamic therapy)) OR (Copper-Cysteamine nanoparticles AND (Reactive oxygen species OR ROS OR Singlet oxygen)). Studies were excluded if they belonged in the categories (a) narrative or systematic reviews, (b) book or book chapter, (c) erratum, (d) short survey, or (e) conference paper. The PRISMA flow diagram illustrates the article selection process (Fig. 4).

## Challenges and future directions

Cu-Cy NPs have emerged as a promising platform for cancer therapy due to their unique ability to generate ROS upon exposure to X-rays, MW, US, and UV light. The resulting oxidative stress induces mitochondrial dysfunction, DNA damage, and pro-apoptotic signaling, ultimately leading to cancer cell death. Preclinical studies have demonstrated encouraging anti-tumor effects, including suppression of tumor growth and metastasis, as well as stimulation of anti-tumor immunity, with generally good biocompatibility and low toxicity in animal models.

Despite the promising preclinical results, translating Cu-Cy NPs into clinical practice remains challenging. Key barriers include differences between animal models and humans, safety and toxicity concerns, and the complexity of the tumor microenvironment. Inefficient targeting and variability in irradiation protocols may lead to inconsistent therapeutic outcomes, while off-target ROS generation raises the risk of damage to healthy tissues. The heterogeneity of cancer types further complicates translation, as tumor-specific ROS sensitivity and microenvironmental factors can significantly alter treatment response.

Addressing these challenges requires a multipronged approach. Rigorous clinical trials are essential to validate preclinical findings and elucidate mechanisms of resistance. Personalized strategies, including the design of smaller and more stable NP formulations, surface functionalization for tumor-specific targeting, and integration with imaging agents or radionuclides, could enhance both efficacy and safety. Optimized irradiation protocols that minimize collateral tissue damage are equally critical. Finally, long-term studies investigating biodistribution, clearance, and copper-related toxicity are needed to ensure safety for clinical application. Together, these measures can help advance Cu-Cy NPs toward becoming a safe and effective theranostic platform in cancer therapy.

## Study limitations

In this review, we summarized in vitro and in vivo studies examining the biological effects of Cu-Cy NPs. A major limitation is the considerable heterogeneity across published studies. In vitro experiments have used multiple stimulation methods, diverse cell models, and varied endpoints. In 16 papers describing in vitro work, the authors used 9 different stimulations, 18 different cellular models, and several endpoints. In vivo studies likewise utilized different xenograft models, animal systems, and irradiation protocols. In 15 papers describing in vivo experiments, the authors applied 11 different stimulations, 12 different xenograft models in 6 different animal systems, and several endpoints. Moreover, these works were not consistent across methodologies. In many cases, only one stimulus was described in the particular experimental model, or similar stimuli differed in the experimental model or endpoint studied. This lack of standardization reduces reproducibility and makes it difficult to draw consistent mechanistic or therapeutic conclusions.

Despite these variations, some consistent responses could be observed across the experiments. In vitro, Cu-Cy NPs induced apoptosis and ROS generation across multiple cancer cell lines, regardless of the type of stimulation applied. In vivo, tumor growth inhibition was reported in nearly all xenograft models, independent of the animal species, tumor type, or irradiation method. These findings suggest a robust anticancer effect, although methodological variability limits definitive conclusions.

Future studies should implement standardized protocols, use more clinically relevant models, and systematically evaluate irradiation conditions. Such efforts will improve reproducibility, enable clearer mechanistic insights, and support the translation of Cu-Cy NPs into clinical applications.

## Conclusion

Despite the challenges and limitations, the promise of Cu-Cy NPs in cancer therapy remains strong. Preclinical studies consistently show anti-cancer effects, including apoptosis induction, ROS generation, and tumor growth inhibition across multiple cellular and animal models. With continued advancements in research and technology, Cu-Cy NPs have the potential to revolutionize cancer treatment by offering more effective, targeted, and personalized therapies. Their ability to combine multiple therapeutic approaches presents an exciting opportunity to improve patient outcomes and enhance quality of life for cancer patients globally. However, careful evaluation through standardized clinical trials is critical to confirm safety, efficacy, and long-term benefits before these NPs can be widely applied in clinical practice.

## Data Availability

No datasets were generated or analysed during the current study.

## Abbreviations

ALT: Alanine aminotransferase
AST: Aspartate aminotransferase
BAX: BCL2-associated X protein
BCL-2: B-cell lymphoma 2
Caspase: Cysteine-dependent, aspartate-specific protease
Cu-Cy NPs: Copper-cysteamine nanoparticles
DSF: Disulfiram (tetraethylthiuram disulfide)
EPR: Enhanced permeability and retention
FRET: Förster resonance energy transfer
FTIR: Fourier-transform infrared spectroscopy
LC3B: Microtubule-associated protein 1 light chain 3 beta
LPO: Lipid peroxide
MDA: Malondialdehyde
MMP: Mitochondrial membrane potential
MW: Microwave
NF-κB: Nuclear factor kappa-light-chain-enhancer of activated B cells
NIR: Near-infrared
NP: Nanoparticle
PDT: Photodynamic therapy
PEG: Polyethylene glycol
PL: Photoluminescence
PLE: Photoluminescence excitation
RES: Reticuloendothelial system
ROS: Reactive oxygen species
SCC: Squamous cell carcinoma
SDT: Sonodynamic therapy
TEM: Transmission electron microscopy
US: Ultrasound
UV: Ultraviolet
UV-VIS: Ultraviolet-Visible
X-PDT: X-ray induced photodynamic therapy
γ-GT: Gamma-glutamyl transferase

## Declarations

### Generative AI in scientific writing

During the preparation of this work the authors used ChatGPT 3.5 in order to improve the writing structure and paraphrase the text. After using this tool, the authors reviewed and edited the content as needed and take full responsibility for the content of the published article.
